# Traumatic brain injury results in acute rarefication of the vascular network

**DOI:** 10.1038/s41598-017-00161-4

**Published:** 2017-03-22

**Authors:** Andre Obenaus, Michelle Ng, Amanda M. Orantes, Eli Kinney-Lang, Faisal Rashid, Mary Hamer, Richard A. DeFazio, Jiping Tang, John H. Zhang, William J. Pearce

**Affiliations:** 1 Department of Pediatrics, Loma Linda University School of Medicine, Loma Linda, CA 92350 USA; 2 Molecular and Integrative Physiology, Loma Linda University, Loma Linda, CA 92350 USA; 3 Physiology and Pharmacology, Loma Linda University School of Medicine, Loma Linda, CA 92350 USA; 4 Anesthesiology, Loma Linda University School of Medicine, Loma Linda, CA 92350 USA; 5 Neurosurgery, Loma Linda University School of Medicine, Loma Linda, CA 92350 USA; 6 University of Michigan, Ann Arbor, MI 48101 USA; 7 Center for Perinatal Biology, Loma Linda University, Loma Linda, CA 92350 USA

## Abstract

The role of the cerebrovascular network and its acute response to TBI is poorly defined and emerging evidence suggests that cerebrovascular reactivity is altered. We explored how cortical vessels are physically altered following TBI using a newly developed technique, vessel painting. We tested our hypothesis that a focal moderate TBI results in global decrements to structural aspects of the vasculature. Rats (naïve, sham-operated, TBI) underwent a moderate controlled cortical impact. Animals underwent vessel painting perfusion to label the entire cortex at 1 day post TBI followed by whole brain axial and coronal images using a wide-field fluorescence microscope. Cortical vessel network characteristics were analyzed for classical angiographic features (junctions, lengths) wherein we observed significant global (both hemispheres) reductions in vessel junctions and vessel lengths of 33% and 22%, respectively. Biological complexity can be quantified using fractal geometric features where we observed that fractal measures were also reduced significantly by 33%, 16% and 13% for kurtosis, peak value frequency and skewness, respectively. Acutely after TBI there is a reduction in vascular network and vascular complexity that are exacerbated at the lesion site and provide structural evidence for the bilateral hemodynamic alterations that have been reported in patients after TBI.

## Introduction

Neurological injuries elicit critical consequences that modify vascular function and can lead to acute and chronic neurological decrements. The role of an abnormal or injured cerebrovascular system in dementia, aging and Alzheimer’s disease as well as stroke have been well characterized^1,2^. In traumatic brain injury (TBI) the importance of the vascular system and its response after injury are ill defined^3,4^. TBI is emerging as a significant public health issue where mild to severe forms of TBI elicit large scale cognitive, emotional and psychological alterations in individuals^5,6^.

Human and rodent vascular networks are extensive with estimates that there is blood supply (i.e. metabolic delivery) within 30 um of each cell in the brain^7,8^. In mouse brain there is ~14 um between neuronal nuclei and the closest vascular supply^8^. Thus even modest alterations in the cerebrovasculature can impart dramatic effects on tissue level metabolic functions which has led some to suggest that hypoxia and ischemic conditions are prevalent in the brain after brain injury^9^.

In patients and in clinically relevant models of TBI acute and chronic alterations in cerebral perfusion have been found. The predominant hemodynamic effects have been acute reductions in cerebral blood flow that often slowly recover over days to weeks^10,11^. However, other vascular features such as cerebrovascular reactivity remain altered long-term^12^. The morphological underpinnings that provide the basis for the modified cerebrovasculature after TBI have not been well described. In patients with severe TBI, post-mortem tissue analysis revealed collapsed, truncated and reduced vascular elements^13^. Similarly, reduced vascular density during the acute phase in moderate and severe animal models of TBI results in functional decrements^10,14^. However, how the vascular network and its cytoarchitecture responds following TBI remains unknown. Interestingly, cerebral blood flow (CBF) is acutely hampered after moderate/severe TBI but then slowly returns to physiological levels^10,15^, but is dependent upon where CBF is measured (peri-contusional vs. distant regions from injury).

Questions about how vessel structure and morphology are altered during the acute period following TBI have not been addressed adequately. This dearth of information hampers efforts to remediate functional vascular decrements reported after TBI. Thus, we hypothesized that a focal moderate TBI elicits loss of vascular structure and complexity. A variety of methods exist to study the intact rodent vasculature^8,14,16^. To test our hypothesis we utilized a novel murine approach that stains the vasculature using the lipophilic 1,1′-dioctadecyl-3,3,3′,3′-tetramethylindocarbocyanine perchlorate (DiI) and modified it for the adult rat to investigate the acute effects of a moderate TBI. We quantified the morphology of the cortical vasculature of the entire bilateral hemispheres using classical angiographic methods. We also utilized novel fractal measures of complexity to evaluate the injured vessel networks that provide unique and complimentary measure of vascularity. Herein, we report that at one day after a moderate TBI there are global reductions in vascular structure alongside decrements in network complexity. Similar but more dramatic reductions were observed at the injury site. Thus, a global reduction in the vascular network during the acute phase (one day) after a focal TBI forms the basis for the acute functional decrements in blood flow and hypoxic conditions that are reported in human patients and in rat models of TBI.

## Results

### Vessel Painting of TBI cortex

**Figure 1:**
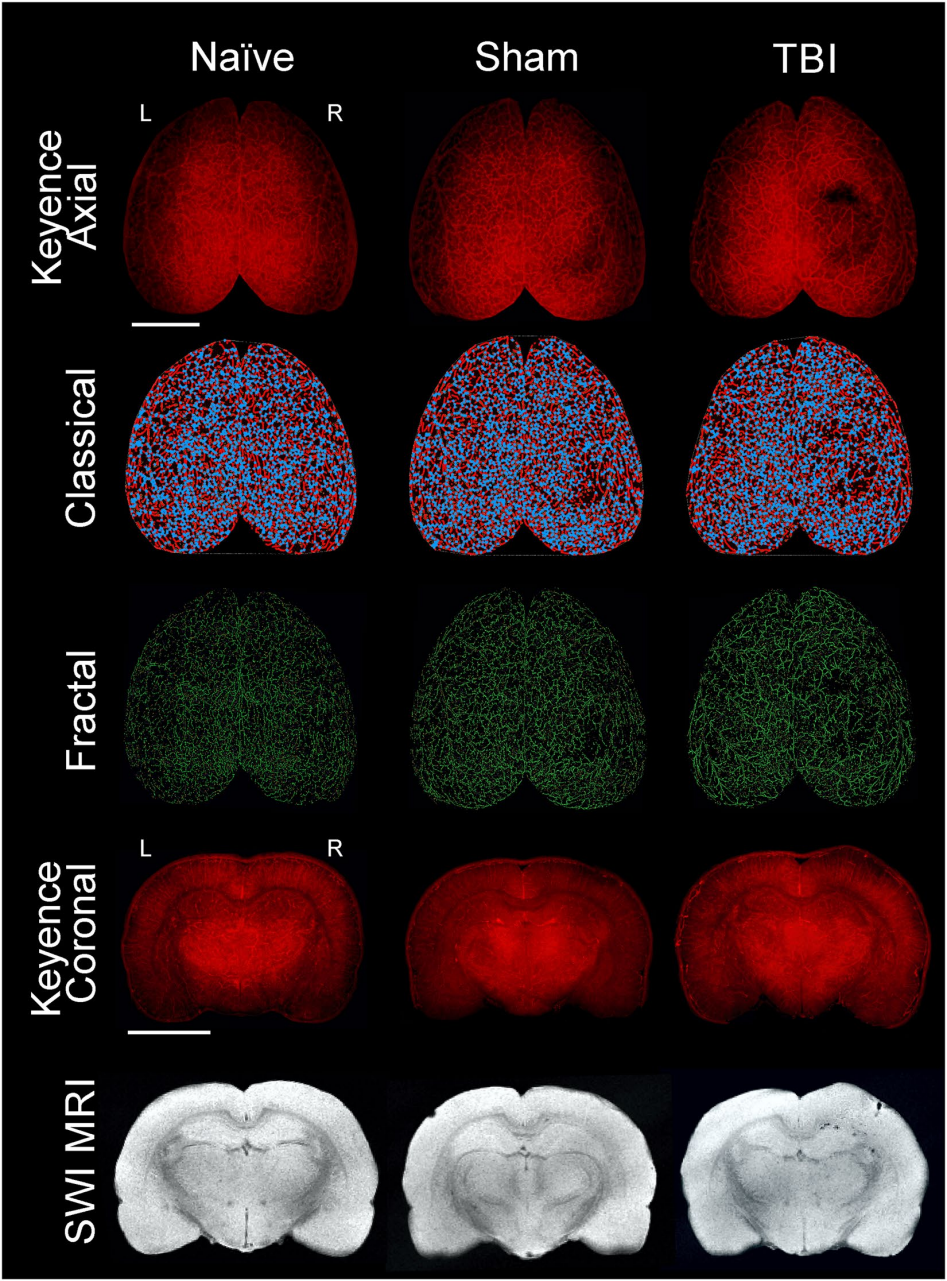
Traumatic brain injury results in vascular loss. The top three rows display representative axial images from wide field epifluorescent vessel painting, classical vascular analysis (AngioTool), and fractal analysis (FracLac) maps. The bottom two rows illustrate coronal images of vessel painted brain tissue and complimentary susceptibility weighted magnetic resonance imaging (SWI MRI) to monitor the extent of extravascular bleeding. Each column is representative for naïve, sham, and TBI brains. The images from the naïve animal demonstrate a diffuse and uniform pattern of vessels without vascular disruptions. The sham animal exhibits slight vascular alterations due to the craniotomy. In contrast, the TBI animal has a clear and overt a region of vessel disruption at the site of injury on both the axial and coronal vessel painted microscopic images. These disruptions to the vascular system after TBI are also visible on the classical vascular analysis maps and on the fractal analysis images.

We report for the first time an adaption of a vessel painting protocol to rats. Previously a similar protocol was developed for mice (Hughes 2014). In our hands, vessel painting of naïve, sham and TBI animals at 1 day post injury (dpi) was successful in 74% of the animals where there was uniform staining (Fig. 1). All brains in the axial view had excellent definition of all surface cortical vessels. Visual inspection of the wide field microscopic images revealed that TBI animals have significant loss of vascular components at the site of the injury encompassing the 3 mm impact zone (Fig. 1, Axial). Subsequently, coronal sections confirmed the depth of the vascular abnormalities in the TBI animals as revealed by the paucity of cortical vessels at the impact zone in the right cortex (Fig. 1, Coronal). No such decrements were observed in naïve animals. Sham animals infrequently exhibited minor vascular alterations (e.g. disrupted vessels at the craniotomy site) and were included in the study.

**Figure 2:**
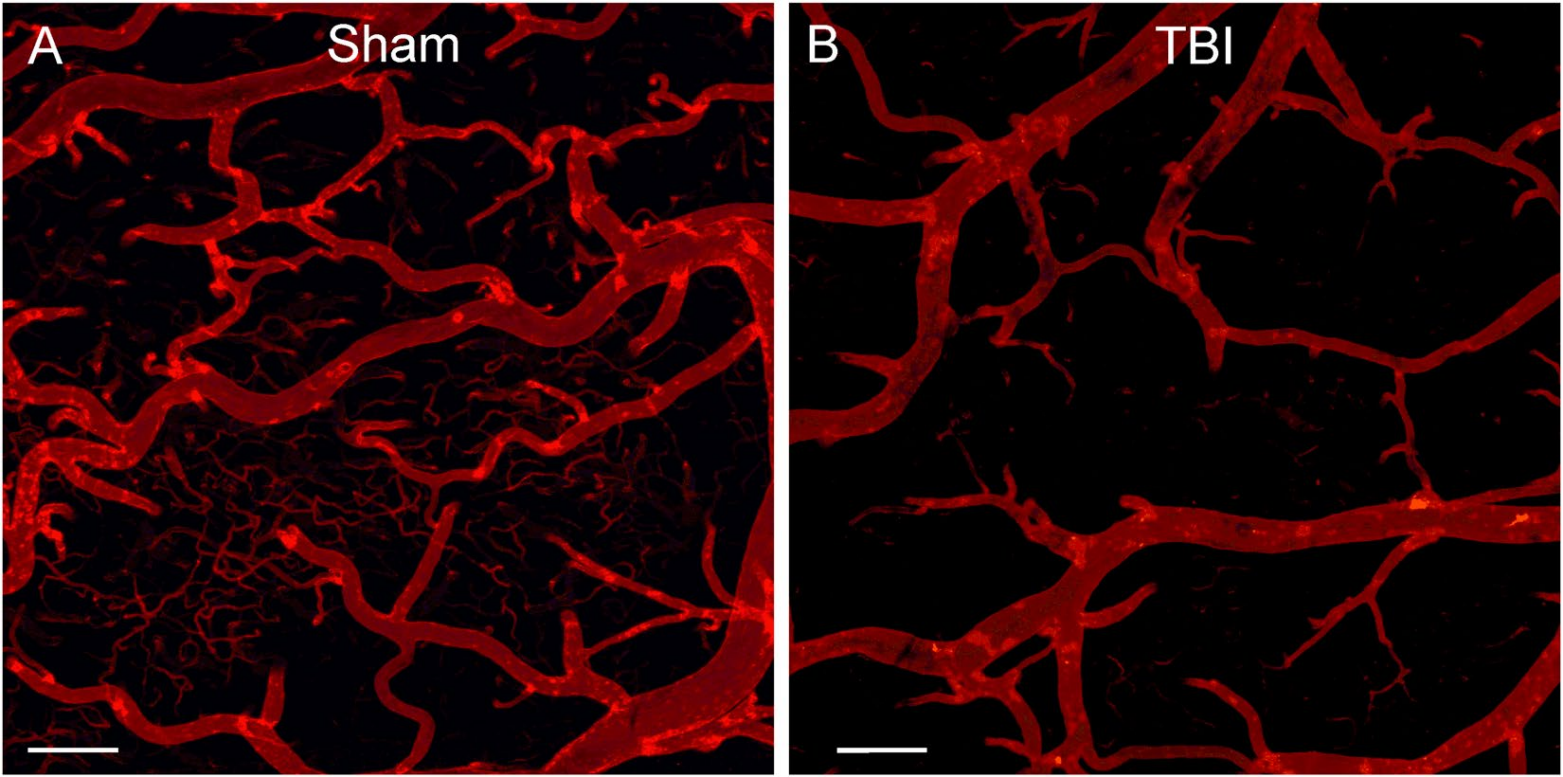
Confocal images of vessel painted vasculature at the perilesional region. (**A**) Sham animal exhibited uniform staining of large, intermediate and small vessels at the perimeter of the craniotomy site. (**B**) The vessels of a TBI animal demonstrate marked sparsity in vessel numbers and dramatic loss of small vessels adjacent to the injury site. Cal bar = 200 um.

Confocal imaging of vessel painted brain tissues in the regions adjacent to the injury site (perilesional) exhibited significant loss of small and intermediate vessels compared to shams (Fig. 2). Thus, vessel painting of the TBI brain can accurately report on the status of the vascular networks, providing a unique window to vessel alterations at local and global scales.

### Classical vascular analysis

**Figure 3:**
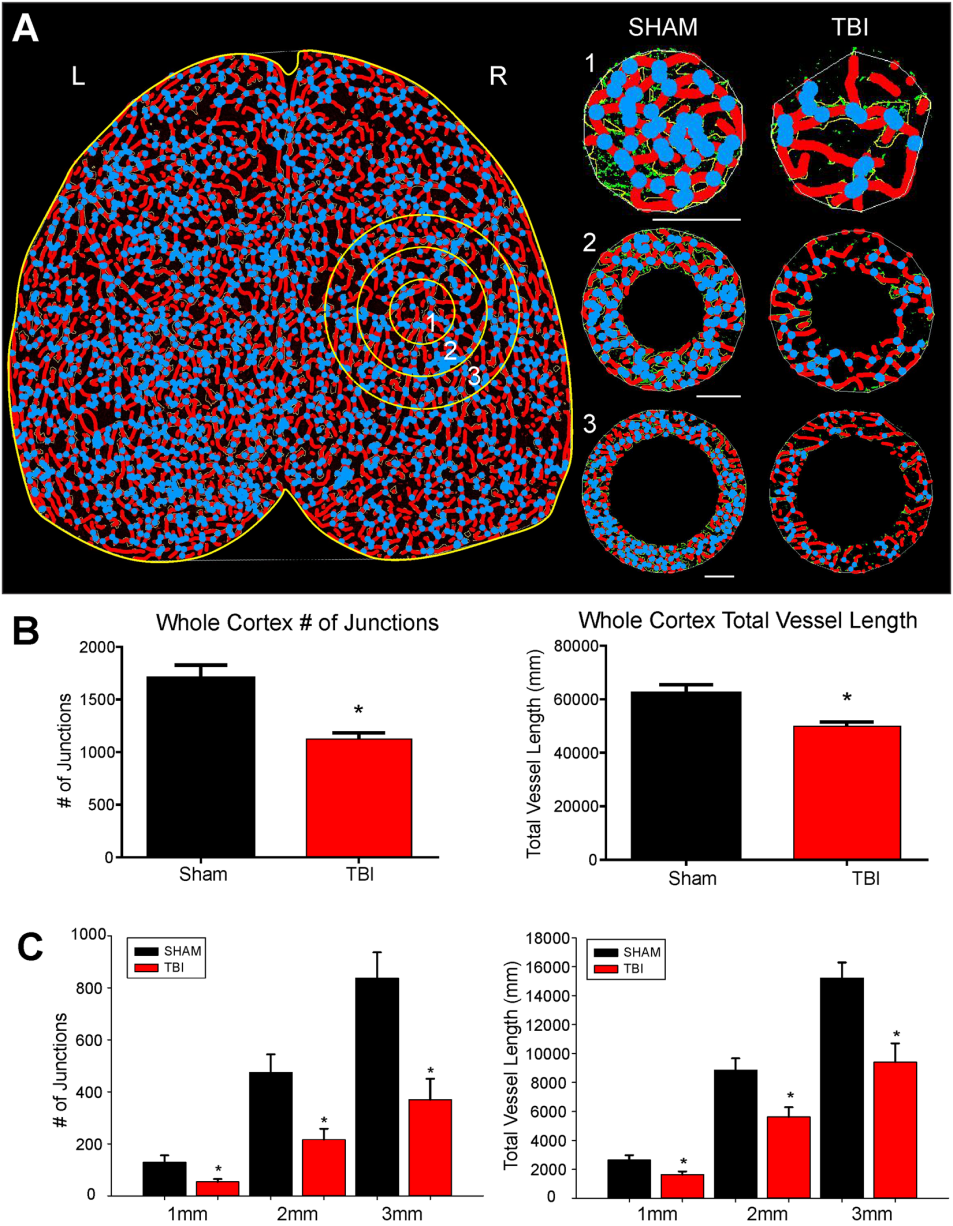
Classical vascular analysis reveals a decrease in the number of junctions and total vessel length following TBI. (**A**) An axial AngioTool image where vessels (red) and junctions (blue) are displayed. Whole cortex and specific concentric radial ROIs projecting outward from the injury site (circles 1–3), were analyzed to quantify vascular alterations. TBI animals exhibited a decrease in the number of vessel junctions and length, even moving outward from the site of injury (1–3 mm). (**B**) Analysis of the entire whole cortex demonstrated a significant reduction in the both number of junctions (t-test, p < 0.05) and in the total vessel length (t-test, p < 0.05) in TBI animals compared to sham animals. (**C**) TBI animals also exhibited a significant decline in the number vascular junctions moving radially outward from the injury site (ROIs 1 to 3) (t-test, p < 0.05). A similar finding was also found in TBI animals where a significant decline in total vessel length moved radially outward from the injury site compared to sham animals (t-test, p < 0.05).

Quantitative analysis of vessel parameters such as branch points, junctions, length and vessel thickness are reported for simple vascular systems such as retinal explants^17^. Recently, an optimized tool for such simple systems was developed and validated for retinal explants (AngioTool)^18^. We now for the first time demonstrate that AngioTool can be utilized for large scale (whole brain) vascular analysis (Fig. 3).

We started with whole brain (both hemispheres) quantification of vessel parameters. The number of vessel junctions for whole cortical slab in TBI animals was significantly decreased by 34.4% (p < 0.05) compared to sham animals (Fig. 3B). Sham animals had a total of 3239.60 ± 95.5 (mean ± SEM) junctions in the entire axial cortical slab compared to 2124.67 ± 79.58 junctions in TBI animals. Similarly, whole brain vessel length (total length of vessels) in the axial cortex was significantly reduced by 21.5% in TBI animals compared to sham animals (p < 0.05) (Fig. 3B). TBI animals 1 day after injury exhibited 95.96 ± 2.00 (×10^−3^) um total vessel length compared to shams that had a total length of 122.31 ± 2.87 (×10^−3^) um.

We further examined hemispheric vessel junction differences and in general found very similar trends between the right (ipsilateral; injured) and left (contralateral, un-injured) hemispheres. TBI reduced the ipsilateral hemisphere by 35.7% compared to shams and the contralateral hemisphere deceased by 33.1% in the total number of vessel junctions (data not shown). A similar trend was found also for vessel length wherein, TBI had reduced vessel length in the ipsilateral hemisphere of 22.3% compared to shams while the contralateral hemisphere exhibited a 20.8% decrease in the total vessel length. When we compared hemispheric differences of the number of vessel junctions between ipsilateral and contralateral axial brain regions in shams we found a 5.2% decrease in the ipsilateral hemisphere and only a modest 1.0% decrease in the contralateral hemisphere in TBI animals. Similarly, for vessel length between hemispheres within each experimental groups we observed a 3.6% decrease in shams between ipsi- and contra-lateral hemispheres but only a 1.7% reduction in TBI animals.

Vessel density was derived and followed a similar pattern as number of vessel junctions and vessel length. Whole brain vessel density was reduced by 26% in TBI animals compared to shams (sham: 115.83 ± 2.54 (×10^−4^), TBI 85.75 ± 2.21 (×10^−4^) um^2^). Hemispheric differences between ipsilateral and contralateral regions also revealed a 6.0% decrease in the sham cortex but only a 0.3% difference between hemispheres in the TBI animals for vessel density. These combined results (vessel length and density, number of vessel junctions) strongly suggested a global reduction in the vasculature across both hemispheres in TBI animals not observed in shams.

To further examine effect of cortical contusions on the vascular networks, we analyzed circular regions moving radially outward from the contusion site. In 1mm increments outward from the epicenter of the injury we observed significant reductions of 58.3%, 54.4%, and 55.8% (1, 2, 3 mm rings, p < 0.05) in the number of junctions compared to shams (Fig. 3C). Similar significant decrements were also found in vessel length with 38.3%, 36.5%, 38.1% (1, 2, 3 mm rings, p < 0.05) in TBI animals compared to shams (Fig. 3C). Thus, all classical vascular parameters analyzed reported significant reductions at 1d after TBI. Further, the reductions in the vasculature after TBI occurred globally in both hemispheres in contrast to sham animals.

### Fractal analysis of the vascular network

Our novel vessel painting approach provided images that reflect a dense vascular plexus that can be difficult to analyze and interpret using conventional methods. Here we chose to use fractal analyzes of the vessel painted brain to provide unique information that cannot be derived from classical vascular analytical tools. We utilized several key fractal features: the local fractal dimension (LFD), skewness and kurtosis.

**Figure 4:**
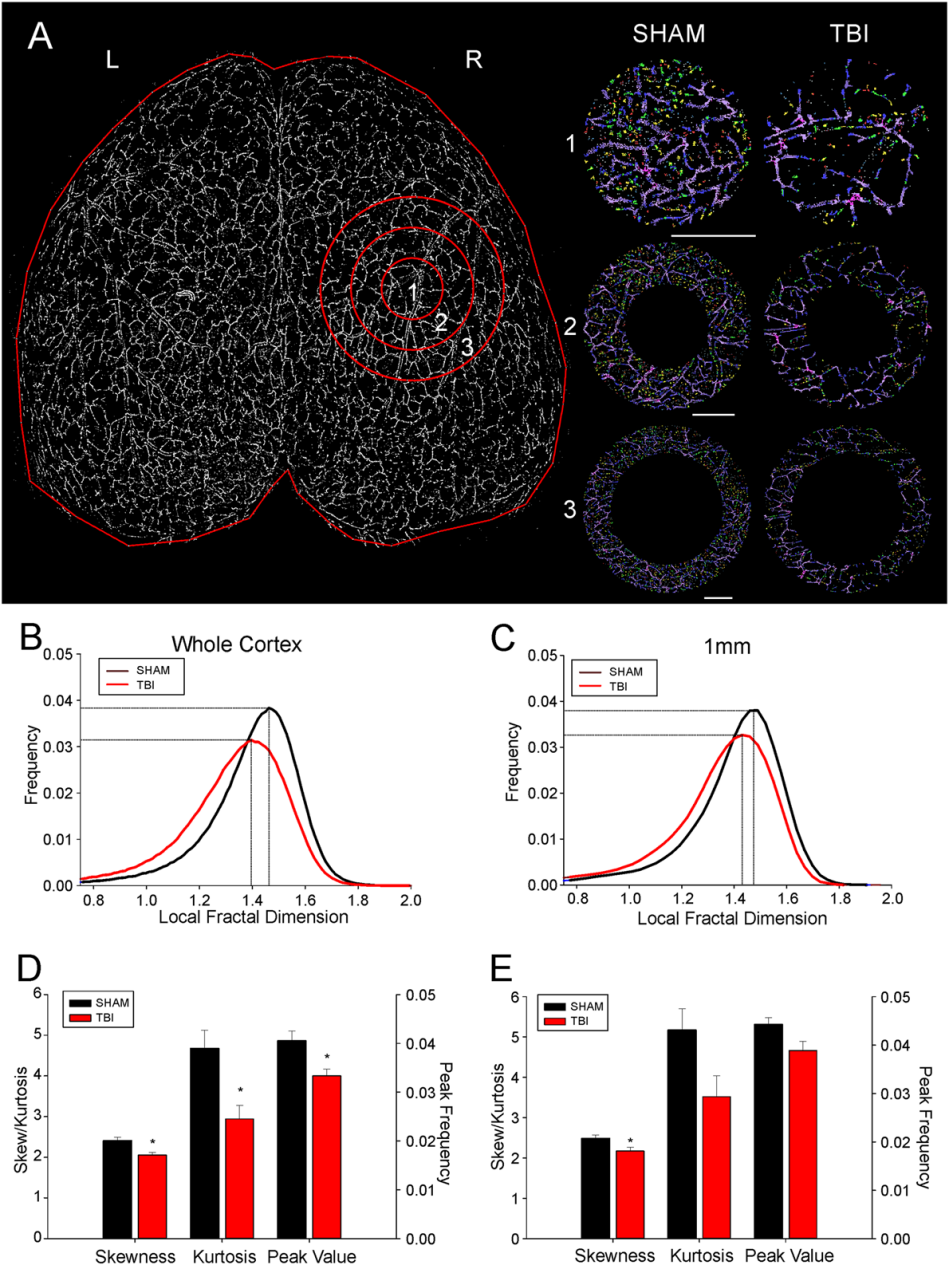
Fractal analysis reveals a quantitative reduction in both vascular complexity and frequency in TBI animals. (**A**) A binary image of the axial vascular network of a representative sham animal with radial ROIs radiating outward from the injury or sham surgery site (ROI1–3). The right panel illustrates the complexity changes in the vasculature from the concentric circles as you move radially outward from the injury site. These fractal images are colorized based on the resultant fractal dimension with a gradient from lower local fractal dimension (LFD) in red (less complex network) to higher LFD in purple (more complex network). (**B**) Histogram distribution of the LFD and peak frequency from the whole cortex of TBI animals reveals a decrease in complexity of the vascular network (leftward LFD shift) along with a corresponding decrease in the peak frequency value (decreased numbers). (**C**) Distribution of the LFD at the injury site (ROI 1) mimics what is observed within the whole brain analysis. At the injury site there is a reduction in the complexity of the vascular networks (leftward LFD shift) along with a concomitant decrease in amplitude of the LFD histogram signifying decreased numbers of vascular components. (**D**) Quantitative analysis of the distribution of LFD histogram from the whole cortex revealed a significant reduction in skewness (t-test, p < 0.03), kurtosis (t-test, p < 0.03) and peak value frequency (t-test, p < 0.03) in TBI animals. The significant change in skewness and kurtosis quantitates the degree to which the complexity of vasculature has been simplified (reduced skewness) and diminished (reduced kurtosis). The peak frequency value synthesizes information on the vascular network and its relative complexity. (**E**) Quantitative analysis of the distribution of LFD at the injury site (ROI 1) reveals similar finding as seen from whole cortex analysis. Significant differences between TBI and sham animals were found only in skewness (t-test, p < 0.05) and not kurtosis or peak frequency values (t-test, p < 0.06).

Whole brain analysis of the LFD found a significant reduction in complexity (Fig. 4A) that yielded a leftward shift of the histograms from shams compared to TBI animals (Fig. 4B) from 1.470 to 1.410 (local fractal dimension, LFD, unitless) signifying a 2.3% change. Also the peak frequency value, a measure of the number of vessels was significantly reduced from 0.038 in shams to 0.031 (LFD) in TBI animals. This 16% decrease in peak frequency represents the number of vessels that are significantly different between groups (p < 0.05). In TBI animals no correlation between the loss of vessel number (peak frequency) and LFD (complexity) could be found (r^2^ = 0.3820, p = 0.19).

When we examined the ipsilateral hemisphere only, we observed a very similar trend: a leftward shift in the LFD, and a decrease in peak frequency (decreased complexity and decrease vessel number, respectively) from the concentric ROIs as they moved radially outward from the impact site (Fig. 4C). At the impact site the peak frequency for shams and TBI animals was 0.040 and 0.034, respectively (p < 0.05). In shams a 1.470 LFD compared to 1.403 in TBI animals was observed. Fractal analyses for both the ipsilateral hemispheric cortex and the impact site revealed a marked decrease in vascular complexity as well as vessel number.

Additional analysis for skewness, kurtosis and the peak LFD for whole cortex (Fig. 4A,D) and for the epicenter of the impact site (1 mm) was undertaken (Fig. 4A,E). For whole brain analysis we observed significant (p < 0.05) reductions in skewness, kurtosis and peak LFD in TBI animals compared to shams (Fig. 4D). Virtually identical trends were found in the 1mm epicenter region where skewness and kurtosis was significantly (p < 0.05) different between shams and TBI animals (Fig. 4E). The peak LFD was reduced in TBI animals relative to shams but not significantly different (p = 0.09).

**Figure 5:**
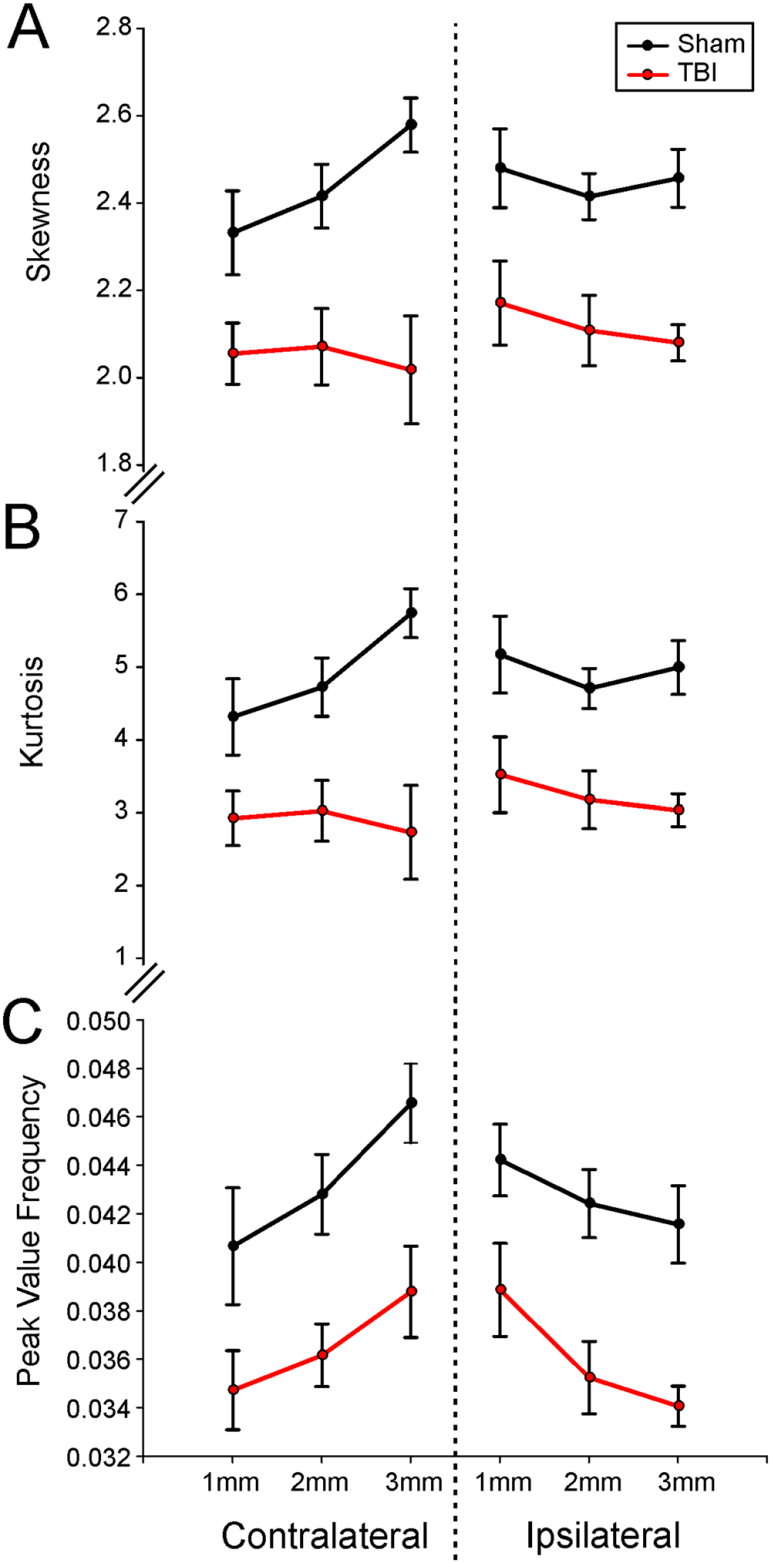
Fractal analyses of the ipsilateral and contralateral brain demonstrate widespread alterations in vasculature complexity and frequency after brain injury. (**A**) The average skewness for radial concentric circles located in the ipsilateral injury site and contralateral corresponding brain region demonstrate a widespread decrease in vascular complexity (skewness) compared to shams. (**B**) Similarly, the average kurtosis from ipsilateral and contralateral brain regions displayed a similar distribution and pattern as found for skewness. (**C**) The average peak frequency value for each concentric circle ipsilateral and contralateral to the injury site confirms a reduction in the complexity of the vasculature in TBI animals compared to shams. Interestingly, the focal injury reductions in the complexity of the vasculature are observed uniformly not only at the ipsilateral injury site but also on identical contralateral brain tissues.

The data from each concentric circle as it moves radially outward from the TBI injury site was acquired as illustrated in Fig. 5. The rationale for performing these analyses examined if vascular structures are differentially affected as you move away from the epicenter of the TBI. All fractal parameters (peak fractal frequency, skewness and kurtosis) investigated in TBI animals exhibited significant reductions on both the ipsilateral and contralateral hemispheres. In general, we found a greater difference between shams and TBI animals at the epicenter compared to the outer rings in the three fractal parameters investigated. Interestingly, the largest changes relative to shams in the ipsilateral hemisphere was found in kurtosis with a 31.9% decrease at the epicenter and a 52.5% decrease in the contralateral hemisphere. The fractal peak frequency also was reduced 11–18% on the ipsilateral side moving radially outward and 16–14% on the contralateral hemisphere. Three important results emerge from these findings: (a) there was a global reduction in vascular complexity (both hemispheres) after TBI, (b) and there was on the ipsilateral side a marked decrease in vascular network parameters that diminish moving outward from the epicenter of the TBI impact site, and (c) these decreases can be seen in all derived fractal parameters. Further confirmation of the utility of using fractals to analyze vascular networks is based on the fact that very similar results can be observed using classical vessel analysis methods (i.e. junctions, vessel length, etc.).

### Magnetic resonance imaging (MRI)

**Figure 6:**
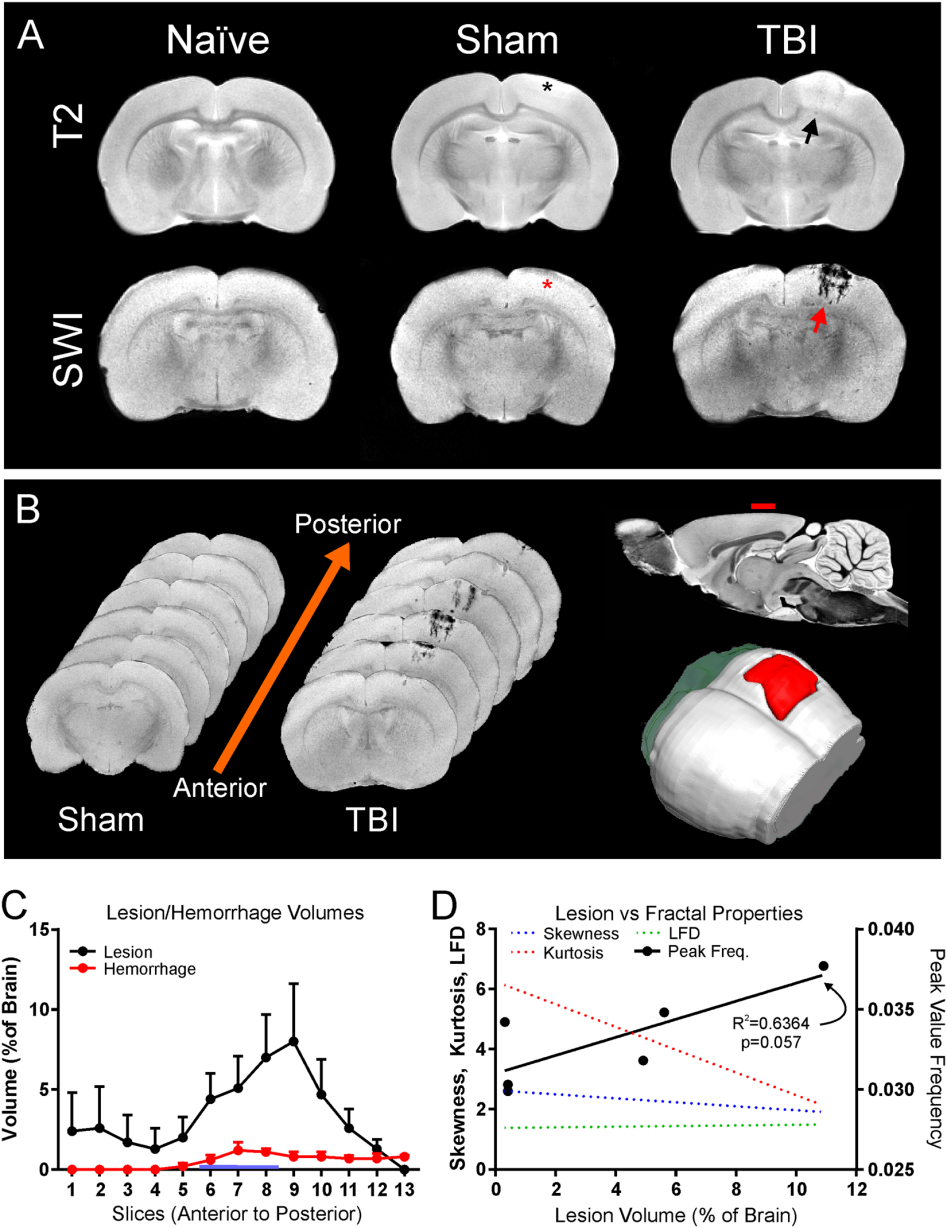
Magnetic Resonance Imaging (MRI) of lesion and hemorrhage volumes in vessel painted brains. (**A**) Representative coronal slices from naïve, sham, and TBI animals at 1d post injury. T2 Weighted Imaging (T2WI) was performed for lesion (blood and edema) volumes while Susceptibility Weighted Imaging (SWI) was used to derive hemorrhage volumes (top and bottom row respectively). Note that the SWI vividly demonstrates hemorrhage in the TBI animal (red arrow) in comparison to the T2WI. Note the lack of bleeding at the craniotomy site in shams (asterisk). (**B**) Coronal SWI images in an anterior to posterior progression from a sham and TBI animal illustrating the anterior-posterior extent of lesion hemorrhage through the cortex (left). Sagittal view of MRI with red dash indicates the site of injury (top-right). A three-dimensional reconstruction from SWI data set shows the expanse of hemorrhage (red, bottom-right). (**C**) Automated lesion and hemorrhage analysis (hierarchical region splitting, HRS) was used to determine the group average from all TBI animals. Edema constituted the largest component of the lesion at 1d after injury. Lesion and hemorrhage volumes were significantly higher in TBI animals when compared to naïve and sham groups (p < 0.05). (**D**) Correlations were performed to determine if individual fractal properties were more predictive of vascular loss and how these were related TBI lesions. Only the fractal peak value correlated to the extent of lesion and approached significance (r^2^ = 0.6364, p = 0.057) but no significance was found for fractal kurtosis (p = 0.093), skewness (p = 0.095) or LFD (p = 0.082).

Confirmatory T2-weighted (T2) MRI was undertaken to assess lesion (edema and hemorrhage) volumes for TBI severity (Fig. 6). Automated lesion analysis revealed total brain injury volume of 3.78% ± 0.01% (% of whole brain) in TBI animals and a small volume of edema was observed in shams due to the craniotomy at 1d post-surgery (1.22% ± 0.007%). Lesion volumes were significantly different from each other (p < 0.0001). Susceptibility-weighted imaging (SWI) was also concurrently acquired due to its exquisite sensitivity to extravascular blood (Fig. 6A,B). We wished to assess if the volume of extravascular blood influenced vascular components. SWI can overestimate the actual volume of blood but this “blooming” effect can be used to detect very small volumes of hemorrhage. We found no significant differences between sham and TBI animals in hemorrhage volume, respectively (0.02% ± 0.02%, 0.27% ± 0.13%, p > 0.05). This lack of significance was partially the result of the heterogeneity of bleeding where 50% of the TBI animals had no MR visible signs of hemorrhage. When we compared only TBI animals with visible hemorrhage versus shams we observed significant differences in hemorrhage volumes (0.02% ± 0.02%, 0.53% ± 0.06%, p < 0.0001).

Additional correlational analyses found only one trend towards a significant correlation between lesion volume and the peak fractal frequency value (Fig. 6D) (r^2^ = 0.6364, p = 0.057). No significant correlations between lesion volumes and kurtosis, LFD or skewness was observed. Similarly there were poor correlations between hemorrhage volumes and kurtosis, skewness, LFD or peak fractal frequency. Thus, in our cohort of TBI animals increased lesion volumes are associated with increased vascular fragmentation.

## Discussion

Neurological injuries are recognized to elicit important consequences that modify vascular function and lead to acute and chronic decrements in neurological function^3^. Acquired and non-acquired disease states such as stroke, Alzheimer’s disease and others are associated with abnormal vascular function^19,20^. While extensive literature exists on stroke and its vascular effects/defects, there are scant reports related to traumatic brain injury (TBI). The first step toward developing putative therapeutics is to develop a comprehensive understanding of the temporal, cellular and molecular events that impact the rodent vasculature following TBI. Therefore, we tested our hypothesis that focal TBI results in significant structural alterations to vascular morphology.

At one day post-injury following a moderate cortical TBI we observed these novel findings: (1) vascular network features were extensively reduced at the site of injury and within the injured hemisphere, (2) while less dramatic than the ipsilateral changes, the contralateral hemispheric vascular features were also significantly reduced, (3) our novel vessel painting approach can sensitively identify vascular alterations, (4) classical vascular analysis methods confirm the observed visual alterations, with significantly decreased junctions and total vascular length, and (5) novel fractal analysis for assaying the complexity of the vasculature after TBI revealed significantly decreased local fractal dimensions (complexity), decreased peak frequency (numbers) along with attendant decreases in skewness and kurtosis of the fractal histogram. Our findings strongly support the concept that the cerebral vasculature after TBI is dramatically altered not only at the injury site but appears to affect brain tissues distant from the TBI (contralateral hemisphere). These alterations in the global vascular network most likely have profound effects on delivery of oxygen and nutrients to both un-injured and injured cortical brain tissues.

TBI induces vascular abnormalities in data from human cadaveric brain tissues^21^ and in rats following fluid-percussion induced TBI^14^. In rats, moderate TBI led to reduced capillary density in the ipsilateral cortex at 1dpi that recovered within 2 weeks^14^. However, while the authors found a recovery in vessel density in the moderate TBI, their findings clearly demonstrate an abnormal “repaired” vasculature^14^. Our findings extend these early observations wherein we report decreased vessel length and vessel junctions along with reduced vascular complexity after TBI. The concurrent reductions in both vessel length and junctions clearly demonstrate that there is a morphological loss and change in the remaining vasculature at 1 day after TBI. One possible explanation for these reductions could be increased fragmentation of the vasculature as a result of the TBI, where larger vessels remain but smaller branching vessels disappear. Such a scenario is supported by our confocal findings in Fig. 2. An important finding of our study was the global modification of morphology of the vascular network throughout both hemispheres. Previous studies have not described global alterations and it is probable that our vessel painting approach was more sensitive in labeling vascular structures. Whether repair or recovery occurs in our TBI model is a question for future studies.

The functional consequences of a reduced or dysfunctional vasculature have been documented previously. In human studies, cerebral blood flow (CBF) was reduced early and continued to exhibit hypoperfusion 14d after TBI^11^. Other vascular findings in TBI patients include loss of autoregulatory capacity^22^, decreased cerebrovascular reactivity^23,24^, and persistent blood brain barrier leakage^25^. Further, in severe human TBI direct observation of the peri-contusional vasculature reported decreased vessel density but no apparent alteration in flow^13^.

Preclinical studies of TBI observed similar vascular dysfunction in a diverse variety of animal models of TBI. A rodent study of moderate TBI, similar to our own, reported decreased laser Doppler flow measures of CBF over the first 3d post injury with a return to control flow levels by 30d after TBI^15^. Using perfusion weighted MR imaging to assess blood flow, Hayward and colleagues observed a triphasic response: early reduced CBF in the ipsilateral cortex at 6 hrs followed by an increase at 24 hrs with a long term depression of CBF from 2 to 14d after injury^10^. Interestingly, the contralateral cortex also had reduced CBF over the first 48 hr after injury. However, blood vessel density assessed by immunohistochemistry was only reduced in the peri-contusional cortex at 14d after TBI with no changes in the contralateral cortex^10^. Others also have reported large (75%) early (1–72 hrs post injury) reductions in CBF in the ipsilateral cortex with a similar time-dependent reduction (45%) in the contralateral cortex^26^. Cerebral blood volume also was reduced following TBI^27^. Clearly, TBI results in early and dramatic perfusion deficits but morphological changes are more variable.

Together, clinical and preclinical studies confirm morphological as well as functional decrements in vascular number and function early after TBI. Several techniques label the vasculature *in situ* including tomato lectin^28^, DiI^16^, Nissl staining^29^ and radiographic methods (computed tomography)^30,31^. We extended development of a mouse DiI approach to rats due to the relative ease of DiI delivery, excellent staining of vascular cellular constituents, ability to perform whole brain wide field fluorescent microscopy and stability of the fluorescent signal over time^16^. We adapted this novel approach to investigate vascular abnormalities throughout the cortex following TBI.

We are the first to report whole brain cortical decrements in vascular features including vessel junctions and vessel length. The reductions, both hemispheric and in those radiating outward from the site of injury, are consistent with previous studies^14,32^. A further novel aspect of our study was the use of intact whole brain samples using classical vessel analysis methods. Previous studies have applied similar tools but only in simplistic vascular models, such as retinal explants^18^. The structural abnormalities we observed are consistent with the morphological alterations to vascular components and cellular structures that have been reported after TBI^10,14^. Rafols and colleagues also found a 40% reduction in the vascular luminal area as early as 6 hrs after TBI that slowly reversed but never fully recovered by 48 hrs post injury^32^. We found a 36% and 23% decrease in vessel junctions and vessel length, respectively, at 1d after TBI within the injured cortex. Our results are in concordance with both morphological and functional changes reported by others where loss of vascular morphology in both hemispheres early after TBI may lead to altered blood flow to brain tissues. Indeed, our findings of a damaged vasculature early after TBI lead us to posit that these changes lead to long-term decrements. As noted above, functional and morphological abnormalities in the vasculature persist after TBI^10,14,21,27^. One can readily speculate that cerebral autoregulation, blood flow and other factors including modifications to elements of the neurovascular unit are altered, perhaps permanently. Clinically, early vascular alterations are thought to lead to long term changes in the blood brain barrier^25^. Finally, vascular reductions and subsequent repair may underlie some of the abnormal connectivity that has been reported using blood oxygen level dependent (BOLD) resting state functional MRI after TBI^33^.

Numerous studies and reviews have shown that the bulk of the neuropathological sequela after TBI may be related to hypoxic and ischemic conditions^9^. Similarly, the impact of post TBI edema can play a significant role in modifying vascular patency. In our present study we did not directly undertake measures of edema. In a CCI model of TBI, Sangiorgi and colleagues concluded that early (3 hrs post TBI) endothelial swelling lead to edema formation^34^. Similarly, in a clinical post mortem study of severe TBI the authors reported that vascular elements were collapsed^21^. Thus, the role edema in TBI and its relationship to morphology of vascular elements warrants closer examination.

Vascular analysis of peripheral tissues, tumor angiogenesis and retinal samples has been accomplished using manual analytic approaches as well as computer assisted measures of vascular density, vascular length and other vessel features^35–37^. Fractal geometric analyses have been used in neuroscience to describe biological complexity^38,39^. Given the complexities of normal healthy vasculature independent of brain injury we used an alternate method for analysis of vessel painted whole brains. Previous studies utilizing fractal analysis of vascular tissues are infrequent and predominantly address retinal or tumor vessel networks^40,41^. Our novel use of fractals was the first to assess vascular complexity in normal and brain injured tissues. We found that there was a global brain-wide reduction in vascular complexity that was more dramatic in the injured hemisphere. The fractal results after TBI are comparable and in concert with the classical angiography analysis methods we performed. Furthermore, while we did not directly measure vascular function (i.e. CBF) our morphological results presented here match those previously published^10,27^. Thus, fractal measures for large scale whole brain vascular morphology are possible similar to those utilized in functional hemodynamic studies^42^.

Non-invasive magnetic resonance imaging (MRI) for brain injury and hemodynamics can assess patient outcomes clinically. We utilized MRI to assess contusional hemorrhage and lesion volumes and found moderate hemorrhage volumes were observed using highly sensitive susceptibility weighted imaging (SWI)^43^. Lesion volumes (edema and blood) were consistent to those previously reported for this model^44^. MRI and vessel painting data provides an opportunity to determine if there was relationship between lesion or hemorrhage volumes and fractal parameters. We report for the first time that peak fractal dimension was positively correlated with lesion volumes, but not hemorrhage volumes. The lack of a correlation with hemorrhage volumes is interesting as hemoglobin breakdown products have been reported to be vasoconstrictive^45^. The finding that fractal dimension correlates better with lesion than with hemorrhage volumes could be because hemorrhage volumes are more variable and perhaps more time dependent, at least in the short term. Fractal dimensions would also have a short-term and a long-term component: vessels die quickly after injury and clot off, whereas new vessels regrow only slowly, and preferentially from very small compared to large metarterioles and arterioles. Contrary to our hypothesis, we found that there was increased peak value frequency (increased vascular components) in TBI animals with larger lesions. One consequence of increased lesion volumes (edema) would be that edema related compression leads to transient preservation of the vasculature. This conclusion garners support in cases of retinal edema with increased fractal dimensions^46,47^. Alternatively, increased peak fractal frequency in cases with increased lesion volume are consistent with potentially increased fragmentation of the vascular network, as might be observed after TBI, particularly with increased lesion volumes. Thus, fractal analysis of whole brain vascular networks correlate with lesion volumes and provide a potential biomarker of altered brain function^48^.

Thus, our findings appear to correlate well with those reported in the literature using MRI assessments of CBF. Work by Grohn and colleagues^10,27^ have provided the most comprehensive *in vivo* imaging of CBF and their studies clearly demonstrated early (<1d) reductions in CBF and cerebral blood volume. Haywood *et al.* did not measure CBF directly at the lesion site but MR perfusion images show reduced CBF at the lesion site^10^. Similarly, Immonen *et al.* also demonstrated reduced blood volume at the injury site^27^. Accordingly, *in vivo* imaging reveals loss of vascular function (CBF) and likely reflects the loss of the vasculature we report using vessel painting.

In conclusion, we have been the first to utilize a novel combinatorial approach for analysis of vascular networks *ex vivo* from the entire cortical surface (cortical slab, 2 mm thick) following TBI. We confirmed our hypothesis where an overall decrease occurs in vascular complexity both globally and at the site of injury. Our classical and fractal analyses converge by finding clear decrements in the vascular network, and are representative of decrements in cerebral blood flow^10^. It is clear that vasculature is altered after TBI. While little work has been done to examine the morphological aspects of the vasculature following TBI, our results and those of others demonstrate that vessels in the brain provide ample opportunities as potential therapeutic targets for ameliorating the deficits observed after TBI. Vessel painting is a novel approach for investigation of the vascular consequences of brain injury from the level of the whole cortex to the individual vessel, ipsilateral versus contralateral, and potentially in a variety of brain injury models. While our current findings examine the vasculature 1d after TBI, ongoing research will address the reported apparent pseudo-normalization of vasculature morphology and function^14^. Finally, nascent findings such as those reported here could be extended to angiographic clinical patient data after TBI as a monitoring tool for cerebrovascular function.

## Methods and Materials

### Animals

All animal experiments and care complied with federal regulations and were approved by the Loma Linda University Animal Health and Safety Committee. Eighteen male Sprague Dawley rats weighing 250–500 g (2–5 months old) were housed in a temperature controlled animal facility on a 12-hour light-dark cycle. Study groups included animals randomly assigned to Naïve, Sham control or TBI groups. Following traumatic brain injury, animals underwent either magnetic resonance imaging (MRI) or vessel painting (n = 5–6/group) and were sacrificed at 1 day post injury.

### Traumatic brain injury (TBI)

The controlled cortical impact (CCI) method used to induce a moderate traumatic brain injury (TBI) has been described previously^49^. Briefly, all animals received a 5 mm craniectomy over the right hemisphere at 3 mm posterior and 3 mm lateral from Bregma whereupon a moderate TBI (4 mm diameter tip, 1.5 mm depth, 5.0 m/s, speed, 200 ms dwell) was delivered to the cortical surface using an electromagnetically driven piston. Sham animals underwent a craniectomy only with no CCI. At the end of each surgery the wound was sutured closed and animals reovered in their home cages. Naïve animals only underwent anesthesia for an identical period of time.

### Animal perfusion and Vessel Painting

Animals were sacrificed via transcranial perfusion at 24 hrs after brain injury. Vessel painting occurs based on the ability of 1,1′-dioctadecyl-3,3,3′,3′-tetramethylindocarbocyanine perchlorate (DiI) to bind directly and preferentially to endothelial cells^16,50,51^. The setup of the perfusion system was critical to successfully labeling the cerebral vasculature along with precise maintenance of perfusion pressures^16^. Care must be taken to remove air bubbles within the tubing and perfusion solution vessels. The Vessel Painting method requires the following sequential order for delivery of perfusion solutions: (a) PBS (150 ml), (b) 50 ml of DiI (13 ug/ml) and (c) paraformaldehyde (4%, 200 ml). After fixation and perfusion, the brain was extracted from the cranium and all meninges removed with care. Photographic verification of the vessel painted brain from the dorsal and ventral views was required to document efficacy of DiI perfusions. Successful vessel painting was defined as uniform staining throughout the cerebrum and animals exhibiting patchy or non-uniform labeling were excluded from the study. In the present cohort incomplete labeling was observed in 67% (6/9) of naïve, 71% (5/7) shams and 83% (5/6) TBI animals. Brains stayed in PBS until MRI or wide-field microscopy (see below).

### MRI Acquisition and Analysis

High resolution MRI was undertaken using an 11.7T Bruker Avance instrument (Bruker Biospin, Billerica, MA, USA) to assess brain injury volumes using two imaging modalities; (a) a standard T2 weighted imaging (T2WI), and (b) a susceptibility weighted imaging (SWI) wherein extravascular blood and hemorrhage are visually enhanced^44^. *Ex vivo* data was collected at room temperature using the parameters in Table 1. The T2 acquired 20 slices for 128 × 128 × 1000 um resolution whereas the SWI images had a total of 40 slices with a resolution of 128 × 128 × 500 um. The primary difference for the resolution between the sequences was a consequence of the acquisition time (Table 1). SWI data was processed to enhance hemorrhage visualization using Signal Processing in NMR (SPIN) software (MRI Institute, Detroit, MI). SWI data was processed using a method similar to that previously described^52^. Briefly, each phase image was processed using a 48 × 48 Hanning filter to remove the low-frequency components, leading to the creation of filtered phase images. Filtered phase data was processed to generate a negative-phase mask that was multiplied into the original magnitude data 4 times to create SWI images.

**Table 1:** MRI Sequence Parameters.

Sequence Type	T2WI	SWI
TR (ms)	2395.9	617.7
TE (ms)	10	7
NEX	2	2
FOV (cm)	2	2
Matrix	2562	2562
Slices (n)	20	40
Slice Thick. (mm)	1	0.5
Slice Int.(mm)	1	0.5
Acq. Time (m:s)	20:26	10:32

Lesion volumes were obtained using HRS, an automated and recursive region segmentation method that segments MR images based on either image intensities or on quantitative MR values (T2 relaxation times, ADC values, SWI phase values, signal intensity, etc), into uniform image regions recursively. In each recursive splitting, regions from the previous step (iteration) were separated into smaller yet more uniform image regions^53^. The HRS method exploited these differences to segment uniform regions in which brighter or darker signal on T2 and SWI maps indicated the location of the brain lesion. HRS extraction of the lesion encompassed: (1) skull stripping; (2) removing background noise; (3) rescaling MR values to reduce computational complexity; (4) modeling the histogram of the rescaled MR values as a bimodal distribution with two distinct and distant peaks; (5) splitting the MR image into two subimages using the valley between these two peaks in the histogram; (6) recursively resplitting the bimodal distributions to generate the HRS tree; (7) stopping the recursive splitting based on a set of uniformity criteria; (8) converting the rescaled values back to actual MR values; and (9) extracting the lesion volume based on ‘a priori’ approximate mean MR values (using a threshold *meanTh*).

### Wide-field Microscopy

Wide-field microscopy used a BZ-9000 Keyence microscope (Keyence Corp, Elmwood Park, NJ). Acquisition of whole brain dorsal and ventral views was performed by carefully positioning the brain between two glass slides with gentle compression to flatten the dorsal surface without damaging the cerebrum. Z-stacks of whole brain fluorescent images at 2× were obtained at each dorsal location using 60 um steps encompassing 50 slices resulting in a 3 mm slab. For coronal images, brain tissue was blocked into 2 mm slices and imaged at 60 um and with 30 slices encompassing 1.8 mm slabs. After acquisition, BZ-II Analyzer software was used to merge dorsal (or coronal) Z-stacks into full focus images. Image processing included haze reduction (Blur/Brightness/Reduction: 10/10/1) to optimize the appearance of vessels.

### Classical Vessel Analysis

Classical vessel analysis included measures of vessel length, thickness, branching, vessel density, and lacunarity. Recent software developments, particularly for angiogenesis, allow for rapid quantitative analysis of vascular networks^18^. AngioTool, an ImageJ plugin, was validated in studies of angiogenesis^18^ and vascular differentiation^17^.

### Fractal Analysis for Vessel Complexity

Fractal geometry for analysis of biological complexity are previously described^38^. Fractal analysis of vascular complexity was calculated using the ImageJ plugin, FracLac^54^. The Local Connected Fractal Dimension (D_LC_) was calculated from binary images at each pixel using the slope of the log regression line for pixel mass against scale. D_LC_ was calculated similar to the mass Fractal Dimension:

$${D}_{Bmass}={\mathrm{lim}}_{\varepsilon \to 0}\lceil \mathrm{ln}({\mu }_{\varepsilon })/\mathrm{ln}(\varepsilon )\rceil $$

where μ_ε_ is the mean pixels per box at some size ε. The calculated pixel mass results from concentrically placed sampling units using a connected set at each pixel to produce a distribution of local variation in complexity in data^54^.

Briefly, each vessel painted image was imported into Fiji image analysis software^55^ for conversion to binary images. The polygon tool was used to outline ROIs. D_LC_ analysis then ensued with FracLac and all output measures recorded and saved. The distribution of D_LC_ (LFD) was then further processed for kurtosis, skewness, peak fractal frequency and local fractal dimension (LFD) for comparisons. Colorized images from the analysis based on the fractal dimension images utilized the default LUT color coding.

The LFD represents patterns at different scales and is used often to represent the complexity of the subject. Skewness and kurtosis of the fractal histogram provide additional measures on how uniformly the fractal dimensions were distributed and how peaked (tailness) the fractal distribution appears, respectively.

### Laser Confocal Microscopy

A Zeiss LSM 710 NLO laser scanning confocal microscope (Jena, Germany) was used to acquire axial images (10× objective lens, wave length Abs: 549 nm, Ems: 565 nm) from the ipsilateral cortex adjacent to the injury site. Z-stack acquisition was obtained at 28.4 um steps and processed for maximum intensity projections using Zeiss software.

### Statistics

All measurements and analyses was performed without knowledge of group. One-way analysis of variance (ANOVA), and Student’s *t-*tests used GraphPad Prism 5.0 (GraphPad, San Diego), followed by Bonferroni’s post-hoc tests when appropriate. Correlation analyses utilized matching data and were corrected using Geisser-Greenhouse correction. Values in the manuscript are presented as mean ± SEM with statistical significance reported at p < 0.05.
